# Rucaparib as a Salvage Treatment in Platinum-Sensitive Relapsed Ovarian Carcinoma: A Case Report

**DOI:** 10.7759/cureus.30405

**Published:** 2022-10-17

**Authors:** Ghanashyam Biswas

**Affiliations:** 1 Medical Oncology, Sparsh Hospital and Critical Care, Bhubaneswar, IND

**Keywords:** platinum senstive replase, parpi, serous ovarian carcinoma, high grade carcinoma, rucaparib

## Abstract

One of the leading causes of female mortality worldwide is ovarian carcinoma. It can be divided into five main types: high-grade serous, endometrioid, clear cell, mucinous and low-grade serous carcinomas. These tumors represent distinct diseases and prognoses. Though the conventional treatment using platinum-based chemotherapy typically results in an adequate initial response, recurrence is not uncommon.

The present case report deals with treating one such platinum-sensitive first relapse of high-grade serous ovarian carcinoma with a poly (ADP-ribose) polymerase (PARP) inhibitor. Here, rucaparib, one of the Food and Drug Administration (FDA)-approved PARP inhibitors, was used to treat platinum-sensitive relapse in a patient with germline breast cancer (BRCA) negative ovarian cancer.

The patient showed a complete response to the treatment and was in remission for around 11 months with only one dose reduction and no blood transfusion. The patient also remained progression-free on regular follow-ups. Since the outcome for this individual case was good, a more extensive study with rucaparib as a second-line treatment option in gene 1 BRCA wild-type homologous recombination deficiency (HRD)-positive ovarian carcinoma patients can be explored.

## Introduction

Ovarian carcinoma is among the most aggressive and recurrent gynecological cancers associated with a poor prognosis and a lack of adequate therapeutic response. Globally, ovarian cancer is the seventh major cause of death in females. In India, just in the year 2012, of the 26,834 women diagnosed with ovarian cancer, approximately 19,549 of them died [1]. In 2020, the crude rate for ovarian cancer in India is 6.4% [2]. As per the Global Cancer Observatory (GLOBOCAN) 2020 data, India accounts for 14.6% of the total world ovarian cancer burden. 

One of the targeted therapies for high-grade serous carcinoma (HGSC) includes a class of poly (ADP-ribose) polymerase (PARP) inhibitors that target the enzyme PARP, which eventually prevents cancerous cells to undergo DNA repair. In the last few years, the Food and Drug Administration (FDA), USA, has approved three PARP inhibitors, namely niraparib, olaparib, and Reucaparib, as maintenance therapy after platinum-based chemotherapy and also as third or fourth-line treatment in breast cancer (BRCA) mutated ovarian carcinomas [3,4]. In the pursuit of this, the present case study deals with treating a 68-year-old post-menopausal female with her first platinum-sensitive relapsed HGSC. The patient was diagnosed with ovarian carcinoma in 2018 and was given platinum-based chemotherapy followed by cytoreductive surgery. After two years of follow-up, the disease relapsed. In 2021, disease recurrence was picked up on a follow-up positron emission tomography-computed tomography (PET/CT) scan and elevated serum cancer antigen 125 (CA125) during the second wave of COVID-19 infection. The patient was offered rucaparib in a low volume even with a negative germline BRCA1/BRCA2 mutation, and the 11 months of follow-up showed no progression.

## Case presentation

The 68-year-old postmenopausal woman presented on July 21, 2018, with a history of abdominal pain and distension for a few days before the presentation. She did not have any comorbidity or family history of malignancy. The ultrasonography and contrast-enhanced computed tomography (CECT) of the abdominal and pelvic regions revealed the presence of a right ovarian mass which was spreading over the omental and peritoneal region.

On July 24, 2018, the PET/CT scan showed a large lobulated complex solid cystic mass in the right adnexal region measuring approximately 5.5 (anteroposterior) x 6.3 (temporal subtraction) x 5.1 ( craniocaudal ) cm in size. The solid component of the mass showed heterogenous post-contrast enhancement and increased fluorodeoxyglucose (FDG) uptake (standardized uptake value (SUV) maximum (max) 8). The right ovary was not separately visualized. Medially the mass was abutting the uterine body and laterally was reaching up to the right lateral pelvic wall. The left ovary measured approximately 1.6 x 1.5 cm in size and showed increased FDG uptake (SUV max 3). The small bowel loops appeared splayed over the mass lesion with no apparent invasion. Inferiorly the right adnexal mass was in close relation to the posterosuperior surface of the urinary bladder. No size significant or FDG avid pelvic lymph nodes were noted. Extensive discrete and coalescent FDG avid (SUV max 14.1) omental and peritoneal deposits involving supracolic and infracolic omentum and the inferior surface of the liver were seen. The omental deposits below the right anterior abdominal wall measured 6.5 x 3.5 cm in size. The serum CA125 was elevated at 1323.35 U/mL. The fine needle aspiration cytology (FNAC) showed adenocarcinoma, and the biopsy showed high-grade serous carcinoma.

**Figure 1:**
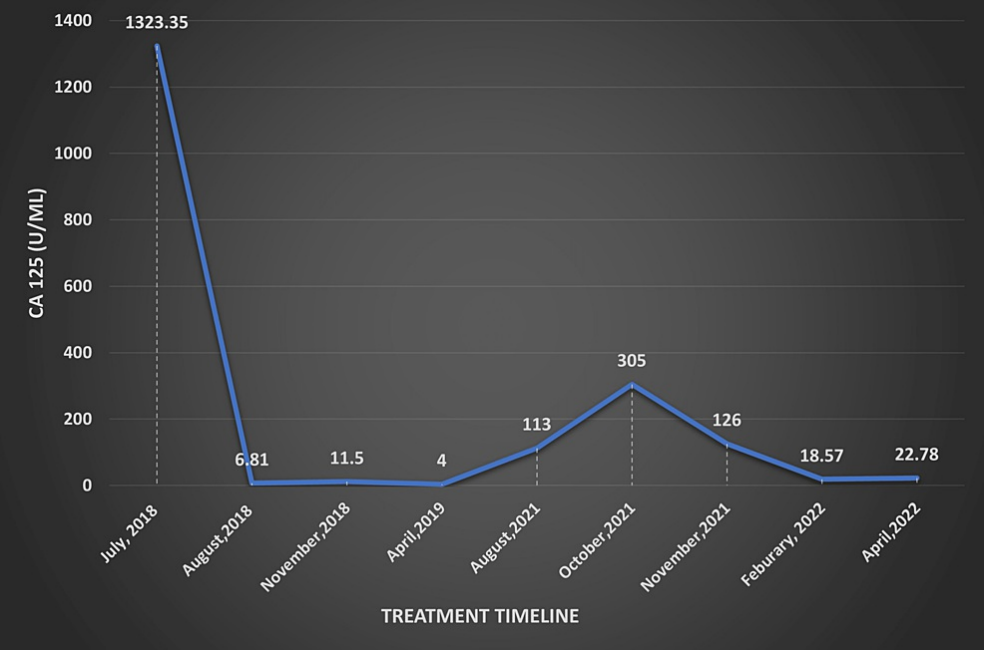
The change in CA125 (U/ml) level during platinum-based chemotherapy and rucaparib treatment CA125: Cancer antigen 125

On August 2, 2018, the patient was started on platinum-based chemotherapy. According to the National Comprehensive Cancer Network (NCCN) guidelines, the patient completed nine doses of a combination of nab-paclitaxel (140 mg/week) plus carboplatin (150 mg/week) over 2.5 months. On November 6, 2018, the PET/CT scan examination revealed a partial response to platinum-based chemotherapy. Mild FDG activity was seen in the enlarged right ovary with a solid cystic/heterogeneous appearance, size 4 X 3.2 cm (`SUV max 3.35, previous 8). The left ovary appeared unremarkable. Multiple small nodes in the retroperitoneum with a max size of 1 cm in the short-axis showed mild FDG activity (SUV max 2.38, previous 5.5). On November 19, 2018, the patient underwent a total abdominal hysterectomy (TAH) with bilateral salpingo-oophorectomy (BSO) with lymph node dissection (LND) and omentectomy. The change in serum CA125 has been illustrated in Figure 1.

 

Follow-up and outcome

**Figure 2:**
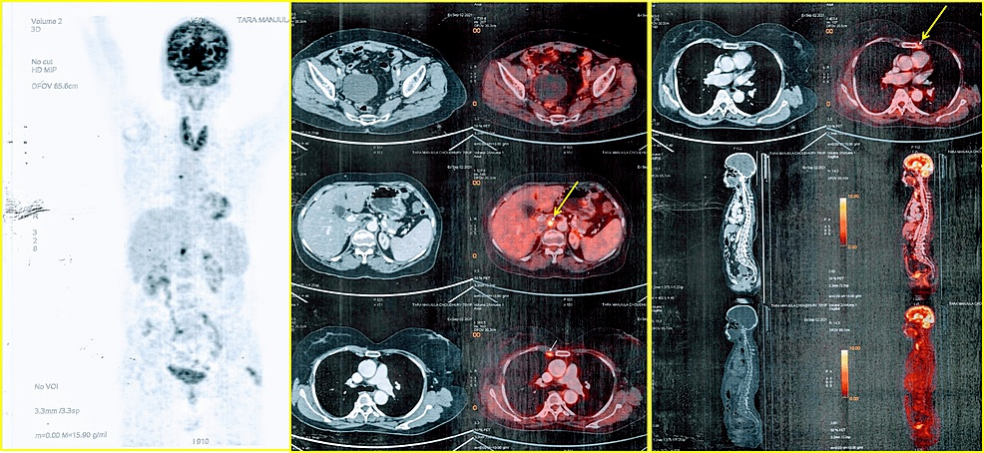
The PET/CT shows a platinum-sensitive relapse of ovarian carcinoma (the arrow indicates sites of abnormal uptake) PET/CT: Positron emission tomography-computed tomography

The two-year follow-up with ultrasonogram (USG) of the abdominal and pelvic regions revealed the absence of disease progression. During follow-up, the germline BRCA1/BRCA2 mutation status came out negative. On August 27, 2021, the patient presented with pain in the extremities and generalized weakness. A PET/CT scan was carried out after an elevated serum CA125 report (113 U/ml). The imaging showed metabolically active aortocaval and bilateral internal mammary nodes, indicating the platinum-sensitive relapse of ovarian carcinoma (Figure 2). The patient also had residual neuropathy from initial chemotherapy.

On September 4, 2021, the patient was started with PARP inhibitor therapy avoiding chemotherapy in view of peripheral neuropathy, and platinum-sensitive radiological and serological relapse in midst of the second covid wave. The patient was started on rucaparib (600 mg tablet x twice daily) which she tolerated well. The five-month treatment with rucaparib showed a profound reduction in serum CA125 levels from 337.7 to 18.57 U/ml.

**Figure 3:**
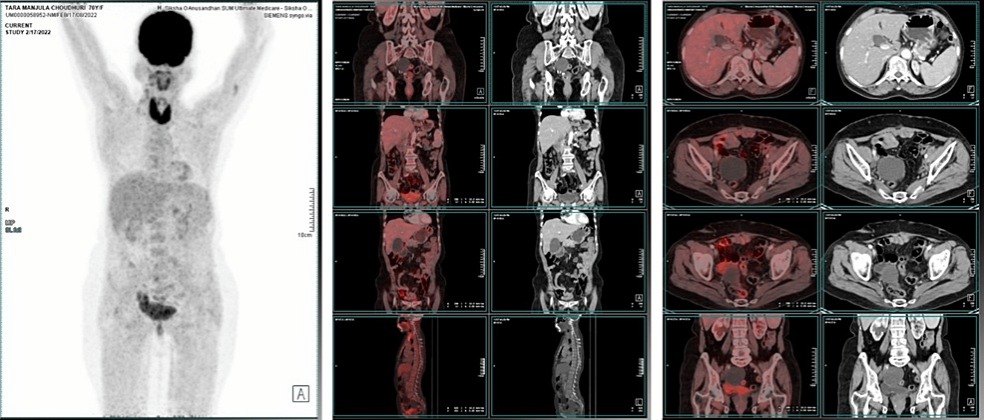
The PET/CT shows a complete response to rucaparib at the sixth-month follow-up PET/CT: Positron emission tomography-computed tomography

**Figure 4:**
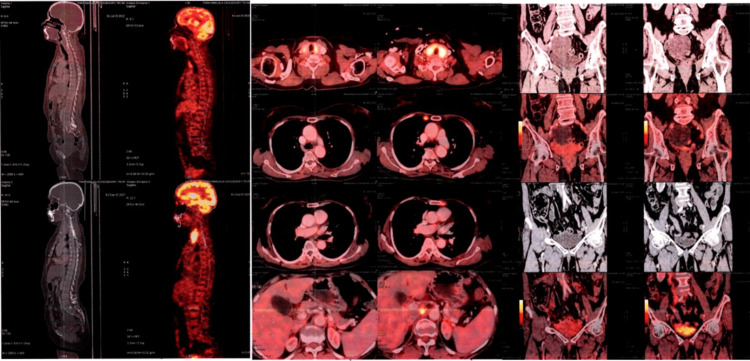
The PET/CT shows a complete response maintained on rucaparib at the 11th-month follow-up PET/CT: Positron emission tomography-computed tomography

On February 17, 2022, the follow-up PET/CT scan findings showed no abnormal metabolic activity in the vault of the vagina or the pelvis. No abnormal hypermetabolic abdominal or pelvic lymph nodes or macroscopic peritoneal disease was noted. There was no evidence of abnormal metabolic activity in the rest of the scanned segments of the body (Figure 3). By observing the PET/CT scan findings, rucaparib was continued. On July 23, 2022, the follow-up PET/CT scan showed similar findings to the previous one (Figure 4). The patient showed a complete response to rucaparib and leads a disease-free life.

## Discussion

High-grade serous carcinoma (HGSC) is associated with a low overall survival rate of 40.7 months as compared to 90.8 months of low-grade serous ovarian cancer [5]. Data from multiple retrospective studies suggest that satisfactory cytoreductive surgery and chemotherapy are the most effective treatments available for HGSC.

Platinum-based chemotherapy combined with surgery is the first-line treatment option for advanced ovarian cancer and its mode of action includes interference with deoxyribonucleic acid (DNA) repair mechanisms that cause DNA damage, and subsequently induce apoptosis in cancer cells [6]. Mostly progression-free survival lasts about 18 months, with a treatment response rate between 60% to 80% [7]. In addition to the good response rate, most patients experience disease recurrence and require second-line or sometimes multiple lines of treatment. Based on the treatment outcome, the disease recurrence can be classified as platinum-sensitive (PFS > six months) or platinum resistance (PFS < six months) [8].

In the present case a 68-year-old, menopausal HGSC patient, was first treated with platinum-based chemotherapy followed by surgery. The treatment was given weekly considering her advanced age, extensive disease, and low serum albumin. The patient lived a disease-free life for two years and then had her first platinum-sensitive relapse during the COVID-19 second wave in India. Platinum sensitivity is a feature of homologous recombination-deficient cells and is evaluated as a surrogate clinical index to predict PARP inhibitor response [9].

As a second-line treatment therapy, rucaparib was initiated post her first platinum-sensitive relapse. The PARP inhibitor-based cancer therapy selectively targets cells with deficient double-stranded (DS)-DNA repair mechanisms like in homologous recombination repair deficiency (HRD) [10-12]. The choice of rucaparib over other PARPi such as olaparib was based on availability and affordability.

The ARIEL-2 trial (multicentre, two-part, phase two, open-label) included 204 patients with BRCA mutant and wild-type BRCA and loss of heterozygosity (LOH)-high platinum-sensitive ovarian carcinomas treated with rucaparib in third-line treatment and beyond, and progression-free survival was longer than in cases with wildtype BRCA LOH-low carcinomas. The results of this trial extend the potential usefulness of PARP inhibitors in treatment settings beyond BRCA mutant tumors [12]. By analyzing both parts of the ARIEL 2 trial, Swisher et al. proposed to consider the administration of rucaparib as an active treatment in earlier lines of therapy before the emergence of platinum resistance [13].

In the present case, the dose of rucaparib was initiated in accordance with the ARIEL-2 trial, which was 600 mg twice daily. The patient experienced severe adverse events including constipation, head reeling/dizziness, and generalized weakness leading to one dose reduction of rucaparib to 900 mg daily. With dose reduction, the patient tolerated the drug better.

Limitations

The present case has the following limitations: 1) the patients have only tested for germline, not somatic BRCA 1/ BRCA 2 mutations; 2: not testing for somatic and BRCA-like mutations may have missed a BRCA effect in the patient; 3) next-generation sequencing of tumor biopsies and quantification of LOH percentage was not done in the patient.

## Conclusions

High-grade serous ovarian carcinoma is known for its recurrence. We did not opt for second-line chemotherapy after the first platinum-sensitive relapse based on various factors such as advanced age, extensive disease, and low serum albumin. Rucaparib was chosen as the second-line treatment due to the COVID-19 pandemic, and the results of rucaparib trials. The outcome for this individual case is good, and the administration of rucaparib has kept the current patient in remission for around 11 months with only one dose reduction and no blood transfusion. Further extensive studies with rucaparib as the second-line treatment option in BRCA wild-type HRD-positive ovarian carcinoma patients are required.
