# Correction: Rucaparib as a Salvage Treatment in Platinum-Sensitive Relapsed Ovarian Carcinoma: A Case Report

**DOI:** 10.7759/cureus.c82

**Published:** 2022-11-17

**Authors:** Ghanashyam Biswas

**Affiliations:** 1 Medical Oncology, Sparsh Hospital and Critical Care, Bhubaneswar, IND

The author's name and affiliation have been corrected. The original published version of this article featured the incorrect spelling of the author's name (as entered by the author) and an incorrect author affiliation (also entered by the author). The name and affiliation have now been corrected. The author deeply regrets these errors.

